# Autoantibodies neutralizing type I interferons underlie a third of cases of Chikungunya virus encephalitis or myelitis

**DOI:** 10.1073/pnas.2616897123

**Published:** 2026-08-17

**Authors:** Vu L. Tran, Adrian Gervais, Marie-Mechtilde Champeaux, Maria Paula de Souza Sampaio, Sylvie Abel, Isabelle Calmont, Mateus Santana do Rosario, Laire Schidlowski, Jordan D. Simione, Pedro Augusto Alves, Anne Puel, Paul Bastard, Laurent Abel, Carolina Prando, Rafael Freitas de Oliveira Franca, Isadora Cristina de Siqueira, André Cabié, Aurélie Cobat, Shen-Ying Zhang, Jean-Laurent Casanova

**Affiliations:** ^a^ St. Giles Laboratory of Human Genetics of Infectious Diseases, The Rockefeller University, New York, NY 10065; ^b^Laboratory of Human Genetics of Infectious Diseases, Necker Branch, INSERM U1163, Necker Hospital for Sick Children, Paris 75015, France; ^c^ Imagine Institute, Paris Cité University, Paris 75015, France; ^d^Laboratório de Investigação em Saúde Global e Doenças Negligenciadas, Instituto Gonçalo Moniz, Fiocruz-Bahia, Salvador 40296-710, Brazil; ^e^Caribbean Clinical Investigation Center, INSERM 2504, University Hospital of Martinique, Fort-de-France 97200, France; ^f^Infectious Diseases and Tropical Medicine Unit, University Hospital of Martinique, Martinique 97200, France; ^g^ Pathogenesis and Control of Chronic and Emerging Infections, University of Montpellier, INSERM, University of the Antilles, Fort-de-France 97200, France; ^h^Faculdades Pequeno Príncipe, Rebouças, Curitiba, Paraná 80230-020, Brazil; ^i^Instituto de Pesquisa Pelé Pequeno Príncipe, Água Verde, Curitiba, Paraná 80250-060, Brazil; ^j^Laboratório de Imunologia de Doenças Virais, Instituto René Rachou, Fiocruz Minas, Belo Horizonte, Minas Gerais 30190-002, Brazil; ^k^Pediatric Hematology-Immunology and Rheumatology Unit, Necker Hospital for Sick Children, Assistance Publique-Hôpitaux de Paris, Paris 75015, France; ^l^Hospital Pequeno Príncipe, Água Verde, Curitiba, Paraná 80250-060, Brazil; ^m^ Plataforma de Pesquisa em Medicina Translacional, Fundação Oswaldo Cruz-Fiocruz São Paulo, Ribeirão Preto, São Paulo 14049-900, Brazil; ^n^HHMI, New York, NY 10065

**Keywords:** Chikungunya virus, encephalitis, myelitis, type I interferons, autoantibodies

## Abstract

Chikungunya virus (CHIKV) infection is typically not life threatening but may, in rare cases, affect the central nervous system (CNS). In three cohorts of confirmed CHIKV cases from Martinique (French overseas territory) and Brazil (patients aged 0 to 89 y, *n* = 245), 20 patients had CNS infection (aged 17 to 87 y). Autoantibodies neutralizing type I interferons (AAN-I-IFN) were found in 35% of patients (4 male and 3 female, aged 17 to 87 y) with encephalitis (4/15), encephalomyelitis (1/2), or myelitis (2/3), but were absent in 225 patients without CNS infection. They neutralized high concentrations (10 to 1,000 ng/mL) of IFN-α2, IFN-α8, and IFN-ω, and the antibodies of one patient also neutralized IFN-β. All samples also neutralized the other 10 IFN-α subtypes (at least 100 pg/mL). This combination of blood autoantibodies occurs in ~0.02% and 0.6% of healthy individuals under and over 70 y of age, respectively. The presence of AAN-I-IFN before CHIKV infection therefore increased the risk of CNS disease ~850-fold relative to the general population.

## Significance

Chikungunya virus (CHIKV) infection results in life-threatening central nervous system (CNS) complications in rare cases. We found that autoantibodies neutralizing type I interferons underlie severe CNS CHIKV infection in 35% of the patients tested. These autoantibodies have previously been shown to underlie encephalitis following live-attenuated CHIKV vaccination and an increasing number of other severe arboviral infections. Screening for such autoantibodies is essential for individuals living in regions endemic for CHIKV and other arboviruses and for devising effective therapeutic strategies for affected patients.

Chikungunya virus (CHIKV) is an *Alphavirus* from the *Togoviridae* family, transmitted to humans primarily by *Aedes aegypti* and *Aedes albopictus* mosquitoes (1). Until the early 2000s, CHIKV caused outbreaks only in Africa and Asia, but its geographic range has since expanded, with notable outbreaks in the Americas, Oceania, and Europe (2, 3). Chikungunya is, therefore, becoming a significant burden on healthcare systems worldwide. CHIKV can cause outbreaks in areas in which no prior immunity has been established, as in the Americas in 2013 (2) and China in 2025 (4). The first locally transmitted cases of CHIKV infection were reported in the United States in 2014 (2), with 3.7 million suspected or confirmed infections occurring in the Americas over the decade that followed (5). Infection rates can be very high, with reports that 10 to 70% of individuals in endemic regions may be infected (6). One recent study estimated the annual number of human infections globally at about 35 million, with most cases occurring in Southeast Asia, Africa, and the Americas (7).

Human CHIKV infection principally causes high fever with polyarthralgia (8). Severe infections are rare, with a fatality rate of ~0.3% in the infected general population (8). In severe cases, CHIKV can lead to life-threatening central nervous system (CNS) infection (encephalitis, meningoencephalitis, myelitis), and/or severe postviral complications of the peripheral nervous system (Guillain–Barré syndrome) (9). The risk of severe acute CHIKV infection requiring hospitalization increases substantially with age and differs between the sexes: The risk of developing severe CHIKV infection in individuals over the age of 60 y is about 20 times higher than that in individuals under the age of 60 y and about 80 times higher than that in individuals aged 20 to 29 y, and the odds of death from CHIKV infection in male patients are twice that in female patients (10). Interestingly, the incidence of CHIKV encephalitis follows a U-shaped distribution with two peaks, one in infancy (<1 y of age) and the other in the elderly (>65 y of age) (11, 12). The mechanisms underlying severe neurological CHIKV in humans remain unclear.

We recently reported that autoantibodies (auto-Abs) neutralizing type I interferons (IFN-I) (AAN-I-IFN) are common and strong determinants of a growing number of life-threatening viral infections (13–19). During the 2025 vaccination campaign in response to the recent CHIKV outbreak on Reunion Island, five unrelated elderly individuals developed severe adverse reactions to the live attenuated vaccine VLA1553 (20). Three had encephalitis and all three had high levels of AAN-I-IFN in blood and cerebral spinal fluid (CSF); the other two had a severely impaired general state without encephalitis and with no detectable AAN-I-IFN. We, thus, hypothesized that AAN-I-IFN might also underlie severe forms of naturally occurring CHIKV infection.

## Results

### Three Cohorts of CHIKV-Infected Individuals.

**Fig. 1.**
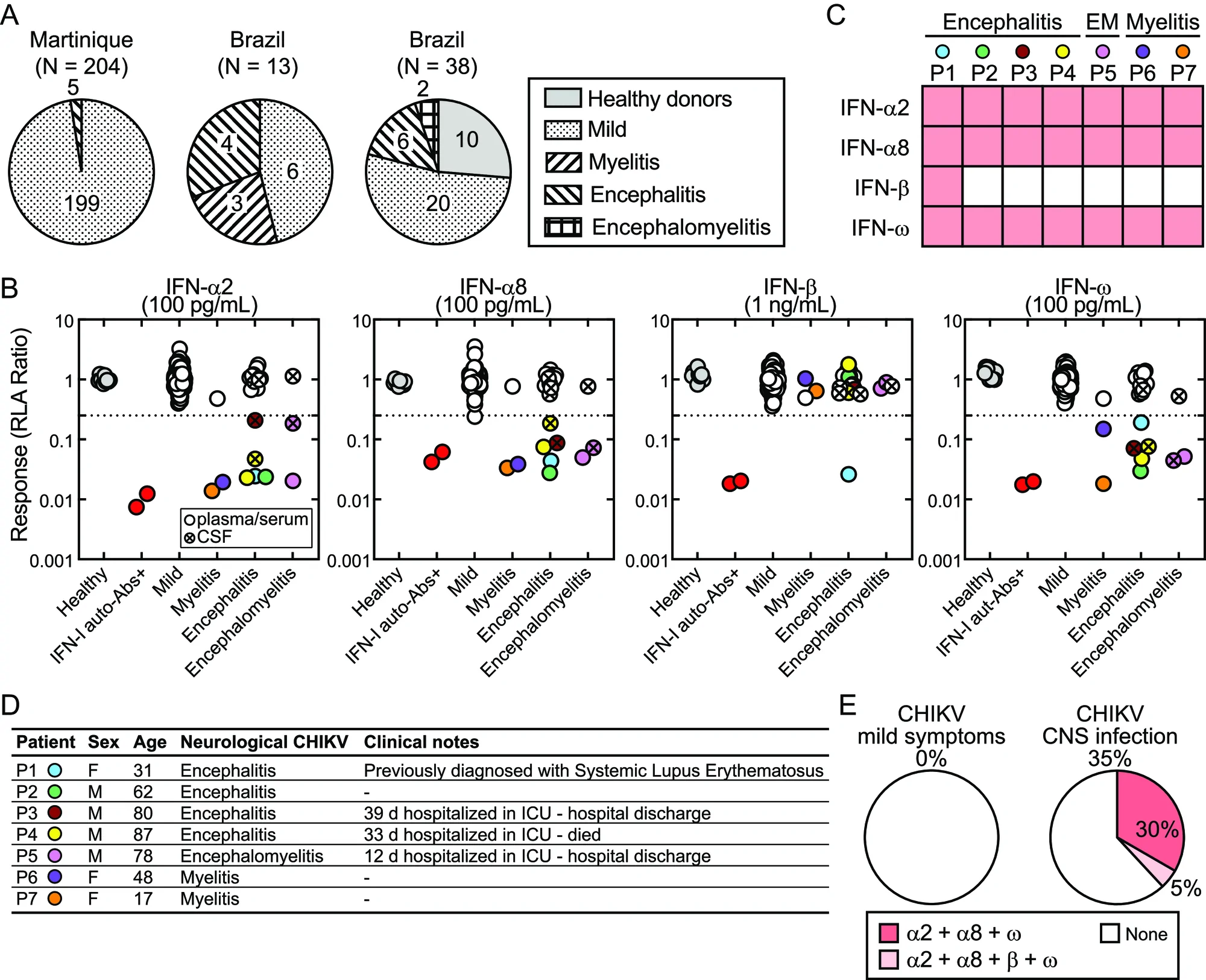
Autoantibodies neutralizing IFN-I in CHIKV-infected patients with severe encephalitis/myelitis. (*A*) 245 CHIKV-infected patients from Martinique (France) and Brazil were classified into different groups on the basis of clinical manifestations. (*B*) Detection of IFN-I auto-Abs in a Luciferase-based neutralization assay; RLA: relative Luciferase activity. Samples with an RLA ratio < 0.25 were considered neutralizing. (*C*) Heatmap depicting the presence of IFN-I auto-Abs in seven individuals. EM: Encephalomyelitis; red: positive, white: negative. (*D*) General clinical characteristics of seven IFN-I auto-Ab-positive patients. ICU: intensive care unit. (*E*) Proportion of IFN-I neutralized in CHIKV-infected patients with neurological complications vs. those with mild symptoms.

We studied 245 individuals with CHIKV infection from Martinique (a French overseas territory, *n* = 204, collected in 2014) and Brazil [two independent cohorts, one from the Northeast, collected from 2014 to 2016 (21), and another from Bahia State in the Northeast, collected from 2020 to 2025 (22), *n* = 41 in total] (Fig. 1*A*). Ten healthy donors from Brazil were included as controls. The CHIKV-infected patients were classified into two different groups based on their clinical manifestations: mild (*n* = 225) and severe CNS infection (*n* = 20 in total), including myelitis (*n* = 3), encephalitis (*n* = 15), and encephalomyelitis (*n* = 2). Most patients with mild symptoms were managed at home without hospitalization. All 20 patients with CNS infection were hospitalized in an intensive care unit (ICU), and one 87-y-old man died after 33 d due to neurological deterioration. Plasma/serum samples were collected from all except four patients during the acute phase of infection [median delay between symptom onset and sampling = 4 d (0 to 31 d)]. We also had access to cerebrospinal fluid (CSF) from six of the encephalitis cases and two of the encephalomyelitis cases from Brazil.

### Autoantibodies Neutralizing IFN-I in CHIKV-Infected Patients with Severe Neurological Complications.

We investigated the presence of AAN-I-IFN in these patients, using a previously described Luciferase-based neutralization assay in A549 IFN Reporter (AIR) cells (23). The neutralization activity of the patient samples served as a surrogate for the presence of AAN-I-IFN. Plasma or serum samples were first tested at a 1:20 dilution for their ability to neutralize 100 pg/mL IFN-α2, IFN-α8, or IFN-ω, or 1 ng/mL IFN-β. Strikingly, six of the 16 patients (37.5%) with severe CNS infection carried circulating AAN-I-IFN effectively neutralizing at least three different types of IFN-I (IFN-α2, IFN-α8, and IFN-ω) (Fig. 1 *B*–*D*). We further tested the CSF (1:20 dilution) of eight patients with severe CNS infection, including two with AAN-I-IFN circulating in the blood, two without circulating AAN-I-IFN and four for whom no serum or plasma was available. Three of the eight patients (37.5%) had CSF AAN-I-IFN, including the two with blood AAN-I-IFN and one of unknown blood AAN-I-IFN status. In total, seven patients carried AAN-I-IFN in blood and/or CSF. They were aged from 17 to 87 y; four were male and three were female. One 31-y-old woman (P1) had previously been diagnosed with Systemic Lupus Erythematosus (SLE), a known etiology of AAN-I-IFN (24), but the other patients were previously healthy. Four of these patients had encephalitis (4 of 15), one had encephalomyelitis (1 of 2), and two had myelitis (2 of 3).

P1 was the only patient whose samples neutralized all types of IFN-I tested (IFN-α2, IFN-α8, IFN-β, and IFN-ω). The samples of three other encephalitis patients (P2-P4) neutralized IFN-α2, IFN-α8, and IFN-ω. Only CSF was available from P3, and it effectively blocked the activity of IFN-α2, IFN-α8, and IFN-ω. Interestingly, both the serum and CSF samples of P4 and P5 displayed neutralizing activity, indicating the circulation of AAN-I-IFN in the CNS, neutralizing IFN-I at concentrations at least as high as 2,000 pg/mL in vivo (equivalent to 100 pg/mL neutralized by samples diluted 1:20). P3 was an 80-y-old man and P4 was an 87-y-old man; both were hospitalized in the ICU, for 39 and 33 d, respectively. P3 was later discharged, but P4 died. P5 was a 78-y-old man; he developed encephalomyelitis and was discharged after 12 d of hospitalization in the ICU. The serum/CSF samples of two female patients with myelitis (P6 and P7) neutralized IFN-α2, IFN-α8, and IFN-ω, but not IFN-β. P6 was 48 y old and P7 was 17 y old. By contrast, none of the 225 cases with mild symptoms had detectable AAN-I-IFN circulating in the blood (Fig. 1*B*). IFN-I auto-Abs were found to underlie a third of severe cases of CHIKV CNS infection (Fig. 1*E*).

### Highly Potent Autoantibodies Neutralizing Supraphysiological Levels of IFN-I.

**Fig. 2.**
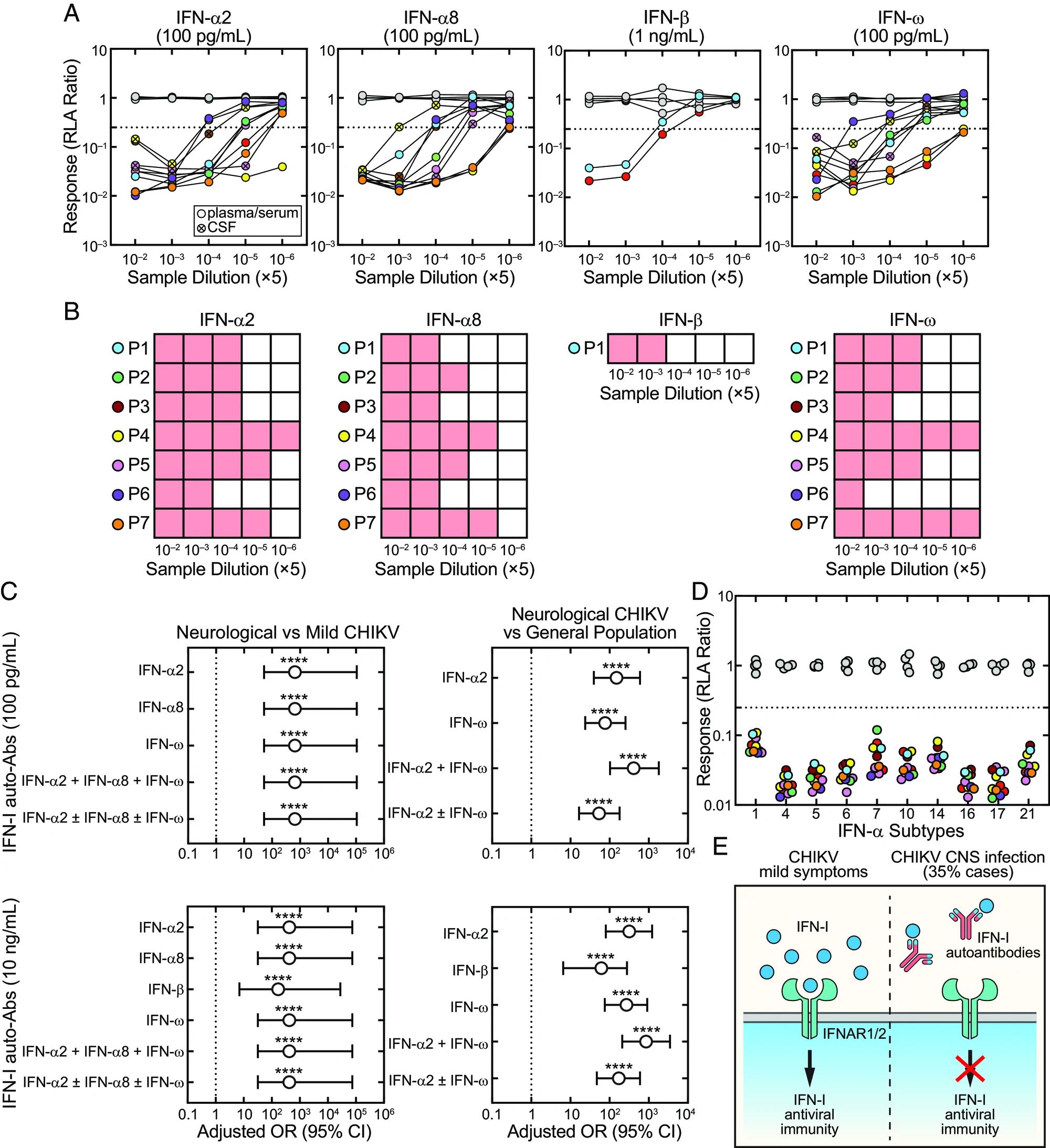
The potency and diversity of IFN-I autoantibodies in patients with severe CHIKV-infection. (*A*) Luciferase-based neutralization assay on serially diluted patient biofluids in the presence of constant amounts of IFN-I; RLA: relative Luciferase activity. Samples with an RLA ratio < 0.25 were considered neutralizing. Gray: healthy donors; Red: IFN-I auto-Ab^+^ controls. (*B*) Heatmap depicting the neutralization activity of diluted samples containing IFN-I auto-Abs; red: positive, white: negative. (*C*) OR for the presence of IFN-I auto-Abs in CHIKV-infected patients with neurological complications relative to patients with milder CHIKV-infection (*Left*) or the general population (*Right*), with adjustment for sex and age. The open circle represents the OR and vertical bars represent the upper and lower limits of the 95% CI. ****P < 0.0001. (*D*) Luciferase-based neutralization assay for auto-Abs against 10 IFN-α subtypes (100 pg/mL). Gray: healthy donors; Red: IFN-I auto-Ab^+^ control. (*E*) Model of auto-Abs neutralizing IFN-I in a subset of CHIKV-infected individuals causing defective antiviral IFN-I immunity and severe clinical outcomes. CHIKV-infected individuals without IFN-I auto-Abs retain normal IFN-I-mediated antiviral immunity.

Seven patients with AAN-I-IFN developed CNS complications of various degrees of severity, ranging from prolonged hospitalization to death. We therefore hypothesized that the patients with the most potent and/or abundant AAN-I-IFN might have more unfavorable outcomes. We then tested all positive samples, serially diluted (from 1:200 to 1:200,000), in the presence of 100 pg/mL IFN-α2, IFN-α8, or IFN-ω or 1 ng/mL IFN-β, to determine the highest equivalent concentration of IFN-I neutralized by these samples. The samples of all patients neutralized IFN-α2 and IFN-α8 when diluted 1:200 (or 5 × 10^−3^) (Fig. 2 *A* and *B*). The samples of P1 neutralized both IFN-α2 and IFN-ω at a 1:2,000 (or 5 × 10^−4^) dilution and both IFN-α8 and IFN-β at a 1:200 dilution. The samples of P6, a patient with myelitis, neutralized both IFN-α2 and IFN-α8 at a 1:200 dilution but was unable to neutralize IFN-ω at this dilution. By contrast, plasma from P7, who had myelitis, retained its neutralization activity even at a dilution of 1:20,000 (or 5 × 10^−5^) for IFN-α2 and IFN-α8 or 1:200,000 (or 5 × 10^−6^) for IFN-ω. Remarkably, the serum of P4 neutralized IFN-α2 and IFN-ω even at a dilution of 1:200,000 and IFN-α8 at a dilution of 1:20,000. This is equivalent to the 1:20 dilution of serum neutralizing 1,000 ng/mL IFN-α2 and IFN-ω and 100 ng/mL IFN-α8. Strikingly, this patient (P4), with the most potently neutralizing auto-Abs of all patients tested, was the only one who died after infection. Interestingly, the CSF of P4 lost its neutralization activity when diluted by a factor of more than 100 times relative to the starter, possibly reflecting the presence of smaller amounts of IgG in the CSF than in plasma. By contrast, both the serum and CSF of P5 retained their neutralization activity when diluted 1:20,000 for IFN-α2 (equivalent of 100 ng/mL IFN-α2 in vivo) or 1:2,000 for IFN-α8 and IFN-ω (equivalent of 10 ng/mL IFN-α8/IFN-ω in vivo). These data indicate that the potency and/or abundance of circulating AAN-I-IFN is not always lower in the CSF than in the blood.

### Risk of CHIKV Disease in Individuals with IFN-I Autoantibodies.

To determine the risk of CHIKV diseases by carrying blood AAN-I-IFN, we estimated the odds ratios (OR) by logistic regression with adjustment for sex and age. Circulating AAN-I-IFN were found exclusively in patients with severe acute CNS CHIKV infection (*P* < 0.0001, Fisher’s exact test), with a risk of severe disease ~400 (OR = 424.4, 95% CI = 32.1 to 74,438.1, *P* = 8.2 × 10^−8^ for 10 ng/mL IFN-I) to ~600 (OR = 653.3, 95% CI = 52.2 to 106,512.8, *P* = 1.6 × 10^−9^ for 100 pg/mL IFN-I) times higher than in patients with milder CHIKV infection (Fig. 2*C*). Previously, we demonstrated that the IFN-α2 and IFN-ω auto-Abs occurs in 0.17% and 1.4% of healthy individuals under and over 70 y of age, respectively, in the general population (25). In this study, neither healthy individuals nor 225 CHIKV-infected patients with mild symptoms carried IFN-I auto-Abs. By using the published dataset (25) as the reference population for comparison, we found that the risk of CHIKV-related neurological complications was about 60 times higher (OR = 61.8, 95% CI = 6.5 to 278.0, *P* = 0.0025) in individuals carrying IFN-β auto-Abs, ~300 times higher (OR = 317.6, 95% CI = 80 to 1,218.0, *P* = 2.2 × 10^−11^) in those with IFN-α2 auto-Abs, ~270 times higher (OR = 269.9, 95% CI = 75.6 to 914.0, *P* = 2.0 × 10^−11^) in those with IFN-ω auto-Abs, and ~850 times higher (OR = 854.6, 95% CI = 209.9 to 3,491.9, *P* = 1.1 × 10^−13^) in the presence of both IFN-α2 and IFN-ω auto-Abs capable of neutralizing 10 ng/mL IFN-I, than in the general population (Fig. 2*C*). The reference population is predominantly of European descent, whereas patients in the current study were from Martinique (Caribbean) and Brazil. Population-level variation in IFN-I autoantibody prevalence across ethnicities has been reported (26, 27), and the true background rate in Caribbean and Brazilian populations is unknown. This difference in ancestry between the current cohort and the reference population is acknowledged as a limitation of the comparison.

### Autoantibodies Neutralizing the 12 IFN-α Subtypes.

IFN-α2 and IFN-α8 belong to the IFN-α family, which contains 12 subtypes, all of which use the same IFNAR1/2 receptor complex for signaling but with different binding affinities and biological potencies (28–31). The biofluids of eight patients were able to neutralize both IFN-α2 and IFN-α8. We therefore tested whether other IFN-α subtypes were also neutralized to a similar extent. We preincubated patient biofluids positive for IFN-α2 and IFN-α8 auto-Abs with 100 pg/mL IFN-α1, IFN-α4, IFN-α5, IFN-α6, IFN-α7, IFN-α10, IFN-α14, IFN-α16, IFN-α17, or IFN-α21 for 1 h before AIR cell treatment for 24 h. All samples effectively neutralized all other members of the IFN-α family (Fig. 2*D*). Interestingly, the neutralization activity of CSF samples from P4 and P5 was similar to that of the corresponding serum for all IFN-α subtypes, consistent with the data previously obtained for diluted samples. These data are consistent with the results of previous studies in which IFN-α auto-Abs, once developed, were shown to neutralize other subtypes of IFNα (14–16, 32).

## Discussion

In conclusion, we report that AAN-I-IFN underlie severe CNS CHIKV infection in ~35% of cases studied. These auto-Abs, with a high neutralization capacity, circulate in both blood and CSF. They effectively neutralize high concentrations (from 1 to 1,000 ng/mL) of IFN-α2, IFN-α8, and IFN-ω, and all IFN-α subtypes in samples diluted 1:20. The auto-Abs were detected in both serum and CSF samples from both patients for which both samples were available, consistent with our previous studies reporting the detection of IFN-I auto-Abs in the CSF of most West Nile virus encephalitis patients with AAN-I-IFN in the blood. We previously showed that AAN-I-IFN are present before severe viral infection and are the cause rather than the consequence of severe viral infection (15, 16, 33). Together with the detection of AAN-I-IFN in patients with encephalitis following live attenuated CHIKV vaccination (20, 34), our findings support a model in which individuals with such auto-Abs are unable to mount a sufficient IFN-I response upon CHIKV infection, thereby rendering them highly susceptible to severe CNS CHIKV disease (Fig. 2*E*). The defective antiviral IFN-I immunity of these individuals may lead to excessive viral replication in the periphery and in target organs, notably resulting in severe CNS infection once CHIKV crosses the blood–brain barrier. Other cases of severe CHIKV CNS infection in individuals without AAN-I-IFN may be due to genetic defects impairing the IFN-I response or production pathways (17, 18).

These findings have important clinical and public health implications. Screening for AAN-I-IFN is warranted in areas in which CHIKV is endemic, particularly for individuals over the age of 65 y. P1 in this study, a 31-y-old patient, was previously diagnosed with SLE, consistent with the finding of AAN-I-IFN in other SLE patients (35), suggesting that screening for AAN-I-IFN in people with known autoimmune conditions might be beneficial. Since P6 and P7 were relatively young and harbored highly potent AAN-I-IFN, it is tempting to speculate that they might have undiagnosed autoimmune diseases. Testing for AAN-I-IFN could be proposed before vaccination with live-attenuated vaccines, including CHIKV VLA1553 (20) and Yellow Fever virus Y17D (36). Such screening program would help identify carriers of AAN-I-IFN in the general population, and AAN-I-IFN carriers should strive to reduce their exposure to ticks and mosquitoes, especially in areas in which arboviruses, such as CHIKV, are endemic. Given the rarity of IFN-β auto-Abs, IFN-β treatment may be helpful for treating or preventing severe CHIKV disease, provided that it is administered early. Other therapeutic strategies potentially appropriate for use in AAN-I-IFN carriers include Chimeric Autoantibody Receptor T cell therapy and decoy IFN-I (37, 38).

## Materials and Methods

### Patients.

We enrolled four independent cohorts of 245 individuals infected with CHIKV from Martinique, France, (CARBO cohort, Clinicaltrials.gov: NCT01099852) and Brazil, and 10 healthy individuals from Brazil. The cases were aged 0 to 89 y; 62% were female and 38% were male. Written informed consent was obtained from each individual in accordance with the local regulations, with institutional review board (IRB) approval. CHIKV infection was diagnosed based on the presence of CHIKV-specific IgM antibodies and/or CHIKV RNA in the plasma, serum, or CSF. Patient stratification was based on the severity of infection and clinical manifestations, including mild symptoms, encephalitis, myelitis, and encephalomyelitis. The full protocol was approved by the IRB committees of the Imagine Institute in Paris, France, and The Rockefeller University in New York.

### IFN-I Neutralization Assay.

We assessed the levels of autoantibodies against IFN-I by neutralization assays, as previously described (23). In brief, 50,000 AIR cells per 100 μL Dulbecco’s Modified Eagle’s Medium (Gibco, 10566016) containing 10% fetal bovine serum and 1% penicillin/streptomycin were dispensed into the wells of a 96-well plate and incubated for 24 h. Patient plasma, serum, or CSF (1:20 dilution) samples were incubated with 100 pg/mL IFN-α2, IFN-α8, or IFN-ω, or 1 ng/mL IFN-β in the presence of EnduRen substrate (Promega, E6482) for 1 h with shaking (600 rpm) at room temperature. Cells were washed with 100 μL phosphate-buffered saline and treated with the sample mixture for 24 h at 37 °C in an incubator with an atmosphere containing 5% CO_2_. *Renilla* Luciferase activity was quantified by measuring the luminescence signal and normalizing against the median of all the samples tested. The 12 IFN-α subtypes were obtained from PBL Assay Science (11002). Samples with a relative Luciferase activity ratio <0.25 were considered to be neutralizing based on the results for a large cohort of healthy donors in previous studies (20, 23).

### Statistical Analysis.

Fisher’s exact test was performed to compare IFN-I auto-Ab levels between the encephalitis/myelitis and the mild infection group. The OR and *P* values for the presence of IFN-I auto-Abs (neutralizing 100 pg/mL for IFN-α2, IFN-α8, and/or IFN-ω or 10 ng/mL for IFN-α2, IFN-α8, IFN-β, and/or IFN-ω) in patients with CHIKV-related neurological complications relative to those with mild CHIKV infection or the general population were estimated by Firth bias-corrected logistic regression, as implemented in the logistf R package, with adjustment for sex and age. Patients for whom plasma or serum samples were tested were included in the analysis. Statistical significance is indicated in the figure legend, as appropriate. **** for *P* < 0.0001.

## Data Availability

All study data are included in the main text.
